# The Medical Treatment of Duodenal Ulcer
*Communicated to the Bristol Medico-Chirurgical Society, November 14th, 1928.


**Published:** 1928

**Authors:** R. C. Clarke

**Affiliations:** Physician to the Bristol Royal Infirmary, the Bristol Orthopædic Hospital, and the Cossham Memorial Hospital, Kingswood


### THE MEDICAL TREATMENT OF DUODENAL ULCER.*

BY

R. C. Clarke, M.B., M.R.C.P.,

*Physician to the Bristol Royal Infirmary, the Bristol Orthopædic Hospital, and the Cossham Memorial Hospital, Kingswood.*

---

The present position of the treatment of duodenal ulcer is a highly controversial one, and there are many schools of thought.

First, there are those who believe that no operative treatment should ever be performed, except for perforation or organic stricture. These are all physicians of the more enlightened type. Then there are those who believe that gastro-enterostomy should be performed after medical treatment has failed. In this category are the majority of physicians and the more enlightened surgeons. Third, there are those who believe that gastro-enterostomy should be performed in every case. These are most of the remainder of the surgeons. Finally, there are those surgeons who, appalled at their post-gastro-enterostomy failures, advocate partial gastrectomy.

It is impossible to go into all the arguments in the matter, and two only are mentioned here. The first is that of the immediate mortality for simple gastro-

* Communicated to the Bristol Medico-Chirurgical Society, November 14th, 1928.

enterostomy. It seems very difficult to get at the figures from any published statistics, but it must be 8 or 9 per cent. if the figures are taken from all over the country. True, some super-surgeons from time to time publish figures as low as 2 per cent., but it must be remembered that these are often selected cases, with a professional anæsthetist and a trained team. At any rate, the victims are not uncommonly met with in the post-mortem room ; and the remark of a pathologist discussing the merits and demerits of his surgical colleagues "that there is nothing wrong with X.'s gastro-enterostomies as they stand a tap pressure in post-mortem," is instructive on this point. If partial gastrectomy were performed on all cases by surgeons the mortality would surely be appalling. Since most cases of duodenal ulcer are in men between 30 and 50, often with dependents, the individual mortality is frequently one charged with grave and even tragic consequences.

The other argument against gastro-enterostomy, though in favour of partial gastrectomy, is the view, at present held by most people, that duodenal ulcer is but a complication of a hyperchlorhydria; a condition easily kept in check by diet and habits, and by no means always cured by gastro-enterostomy. If the hyperchlorhydria persist after gastro-enterostomy, the risk of jejunal ulcer is a heavy one. It is quite impossible to find any reliable statistics as to the frequency of this complication. A surgeon never gets them in his own cases, but seems to see plenty in other surgeons' cases. It is a condition which cannot be cured medically, and can only be relieved by restoring the parts to their natural state.

The principles of medical treatment, which are very well known, were inaugurated by Sippy, and have been developed by various workers since, in England principally by Hurst, Maclean, Ryle and Bennett. They depend upon the facts that in the absence of free hydrochloric acid a duodenal ulcer will heal in from three to six weeks, depending upon its chronicity and depth; and that recurrence can be prevented by a régime which is neither arduous nor difficult, but will keep the hyperchlorhydria in check. Many treatments have been published on these lines. The primary object to start with is to keep the free hydrochloric acid out of the stomach by day and night. This is done by giving milk, bland foods and alkalies. On the value of various alkalies there is much difference of opinion. Hirst has lately published some researches, done by himself and his co-workers, on the efficiency of various alkalies.

The régime the writer has adopted for the last four or five years is as follows:—

*First Treatment.*

| | |
|---|---|
| 6. 0 a.m. | Powder No. 1. |
| 7. 0 ,, | Milk 6 oz. with Mist. No. 1. |
| 7.30 ,, | Powder No. 2. |
| 8. 0 ,, | Custard or Benger's 6 oz., groats or junket with fruit jelly, preceded by Mist. No. 2. |
| 8.30 ,, | Powder No. 2. |
| 9. 0 ,, | Milk 6 oz. with Mist. No. 1. |
| | Repeat the same at two-hourly periods till 9.0 p.m. |
| 9.30 ,, | Mist. No. 2 (double dose). |

Milk with Mist. No. 2 and powder No. 2, given at night twice if awake.

*Pulv. No.* 1.—Bismuth. Carb. dr. i, Magnesii Carb. Pond. grs. xv, omni mane.

*Pulv. No.* 2.—Bismuth. Carb. grs. x, Cret. Prep. grs. xv.

*Mist. No.* 1.—Sodii Citrat. grs. vi, Magnesii Hydroxid. dr. i, Aq. ad. oz. i.

*Mist. No.* 2.—Tr. Bellad. m. v, Aq. ad. oz. i.

*Second Treatment.*

| | |
|---|---|
| 6. 0 a.m. | Milk 6 oz., Powder No. 2. |
| 7. 0 ,, | Breakfast. Egg, bread and butter and milk. Powder 1 oz. an hour after. |
| 8.30 ,, | Lunch. Milk 6 oz. |
| 11. 0 ,, | Milk 6 oz. |
| 12. 0 noon. | Dinner. Ordinary. Powder No. 2 an hour after. |
| 3. 0 p.m. | Tea. Bread and butter, and one egg. Powder 1 oz. |
| 5. 0 ,, | Milk 6 oz. |
| 7. 0 ,, | Benger's, custard. |
| 9. 0 ,, | Milk 6 oz., Powder No. 2. |
| 9.30 ,, | Tr. Bellad. m. xv. |

The first part is continued from three to six weeks after any septic focus, such as infected teeth or a chronically inflamed appendix, has been removed.

The second treatment is carried on with the patient up and at work for another two months, and the patient gradually gets on to a normal life, with the following restrictions :—

*Instructions to patients with duodenal or gastric ulcers.*

1. Never be more than three hours without food.

2. Milk is the best " quick meal " that you can take.

3. Avoid pips and skins in fruit or jam.

4. Thoroughly chew everything before you swallow it.

5. Avoid tough meat, uncooked vegetables, pickle, pepper, mustard and other condiments.

6. Smoke as little as possible.

7. Avoid short drinks and fizzy drinks before meals. Beer or weak whisky and water is the least harmful way of taking alcohol, which must be taken at meal-times.

8. Drink milk, or (if you can) olive oil, the last thing at night.

9. Have your teeth attended to every six months.

10. Keep some powders and medicine, and if you get a return of your symptoms go to bed immediately on a milk diet with the powders.

There are little difficulties encountered in the first part of the treatment. The mouth sometimes becomes dry and uncomfortable, and needs constant attention. Some patients are intolerant of belladonna ; and occasionally constipation is a difficulty. No patients have shown symptoms of alkalosis. Pain disappears almost invariably at the end of forty-eight hours, and recurrence is extremely rare.

It is not suggested that this treatment is by any means the last word or the best ; and it may, of course, be modified slightly to suit individual cases. All that is claimed for it is that it will heal a duodenal ulcer, and that the patient afterwards is in a better position than one who has had a gastro-enterostomy. As to the future of these patients, they are in the same position as those with diabetes or diverticulosis of the large intestine. Only those in whom the condition is undiagnosed or who neglect their after treatment need get into serious trouble.

